# *Sonchus asper* and Its Potential in Cosmetics—A Review

**DOI:** 10.3390/ph19060890

**Published:** 2026-06-04

**Authors:** Dorota Kasprzak, Natalia Dycha, Magdalena Michalak-Tomczyk, Anna Wawruszak, Magdalena Zdziebło, Wirginia Kukula-Koch, Grazyna Ginalska

**Affiliations:** 1 Department of Beauty Sciences, Faculty of Health Sciences, Vincent Pol University, 2 Choiny Str., 20-816 Lublin, Poland; d.kasprzak@pol.edu.pl (D.K.); g.ginal@poczta.onet.pl (G.G.); 2 Department of Pharmacognosy with Medicinal Plants Garden, Medical University of Lublin, 1 Chodzki Str., 20-093 Lublin, Poland; dychanatalia98@gmail.com; 3 Doctoral School, Medical University of Lublin, 7 Chodźki Str., 20-093 Lublin, Poland; 4 Department of Physiology and Toxicology, Faculty of Medicine, The John Paul II Catholic University of Lublin, Konstantynów 1I Str., 20-708 Lublin, Poland; magdalena.michalak@kul.pl; 5 Department of Biochemistry and Molecular Biology, Medical University of Lublin, 1 Chodzki Str., 20-093 Lublin, Poland; anna.wawruszak@umlub.edu.pl; 6 Branch in Sandomierz, Jan Kochanowski University in Kielce, 13a Schinzla Str., 27-600 Sandomierz, Poland; magdalena.zdzieblo@ujk.edu.pl

**Keywords:** cosmetics, prickly sow-thistle, inflammation, skin cancer, antiradical properties, antimicrobials

## Abstract

*Sonchus asper* (L.) Hill is a widely distributed plant traditionally used as both a food source and a medicinal herb. In recent years, increasing interest in natural, safe, and effective cosmetic ingredients has highlighted the potential of plant-derived bioactive compounds. This review provides an overview of the biological properties of *S. asper*, with particular emphasis on its relevance in cosmetic applications. The plant is characterized by a rich profile of primary and secondary metabolites, including amino acids, fatty acids, vitamins, phenolic acids, flavonoids, coumarins, and terpenoids. These compounds contribute to a broad spectrum of biological activities, such as strong free radical scavenging activity, modulation of inflammatory pathways, and inhibition of the growth of selected skin-associated pathogens, suggesting its potential as a multifunctional cosmetic ingredient.

## 1. Introduction

Plants represent a rich and diverse source of bioactive compounds, including polyphenols, alkaloids, terpenoids, and other secondary metabolites, which have long been utilized in traditional medicine as well as in human nutrition. Their broad spectrum of biological activities, including antioxidant, anti-inflammatory, antimicrobial, and metabolic regulatory effects, has contributed to their continued relevance in both pharmaceutical and functional food applications [1].

In recent years, increasing awareness of health, well-being, and skin condition has led to a growing interest in natural products as sources of functional ingredients. This trend has significantly influenced the cosmetic industry, where there is a rising demand for safe, effective, and non-toxic formulations derived from natural sources. Consumers increasingly prefer plant-based ingredients, which are perceived as more biocompatible and environmentally sustainable compared to synthetic compounds [2].

Numerous plant species have already been widely incorporated into cosmetic formulations due to their well-documented biological activities. Examples include *Camellia sinensis*, *Aloe vera*, *Punica granatum*, *Psoralea corylifolia*, and species of the genus *Cistus*, which are commonly used in dermocosmetic products due to their antioxidant, anti-ageing, soothing, and regenerative properties [3].

In this context, there is a growing need to explore less-studied plant species with comparable or complementary biological potential. *Sonchus asper* (L.) Hill also called prickly sow thistle, represents one such species, characterised by a rich phytochemical profile and a broad spectrum of biological activities [4,5]. The aim of this review is to highlight the cosmetic potential of *S. asper*, with particular emphasis on its antioxidant, anti-inflammatory, antimicrobial, and other skin-relevant properties, and to position this species as a promising source of bioactive compounds for future cosmetic applications.

*Sonchus asper* is an erect, robust, spiny annual or biennial herb characterised by a hollow, slightly branched stem and simple, sessile leaves with an auricular base and membranous blade, typically reaching 6–17 cm in length. The plant produces a basal rosette and yellow flowers and may grow up to 1.8 m in height. It is native to Europe but is now widely distributed across the American continent, Africa, Asia, Australia, and New Zealand, occurring in diverse habitats such as cultivated fields, roadsides, wetlands, dunes, and disturbed environments. Traditionally, *S. asper* has been used both as a food and medicinal plant; it is commonly consumed as a wild edible vegetable in salads, soups, and cooked dishes, particularly in Mediterranean regions, and has been employed in folk medicine for the treatment of ailments such as cough, bronchitis, gastrointestinal disorders, wounds, burns, and inflammatory conditions [5,6,7]. Recent findings underline its potential application in cosmetics.

## 2. Prickly Sow Thistle as a Source of Diverse Metabolites

### 2.1. Primary Metabolites

Primary metabolites identified in *Sonchus asper* are predominantly associated with its nutritional value and physiological functions. They include amino acids, lipids, carbohydrates, vitamins, and carotenoids. Recent metabolomic investigations based on LC–MS analysis revealed the presence of six essential amino acids, namely L-lysine, L-threonine, L-isoleucine, L-leucine, L-phenylalanine, and L-tryptophan, confirming the contribution of *S. asper* to nitrogen metabolism and its nutritional relevance. These amino acids constitute key primary metabolites involved in protein biosynthesis and cellular homeostasis [6].

Also, lipids are an important fraction of *S. asper* primary metabolites, with reported concentrations of approximately 1.32 g/100 g fresh weight. Phytochemical analyses further confirm the presence of fatty acids, like linoleic acid (C18:2), or alpha-linolenic acid (C18:3) as key structural and energy-storage molecules, essential components of cellular membranes involved in signalling and metabolic processes [6]. Next to them, several oxidised fatty acids were detected, with trihydroxyoctadecadienoic acid, trihydrozyoctadecenoic acid, hydroxy C18:2 and hydroxy C18:3 fatty acids [6]. Among the metabolites of prickly sow thistle, carbohydrates find their place as well. With an estimated quantity of 0.34 g/100 g fresh weight, they represent another major class of primary metabolites, functioning as energy sources and metabolic intermediates. Moreover, *S. asper* contains multiple vitamins, including vitamins E and K, as well as other micronutrients, which contribute to its antioxidant and nutritional properties. Additionally, ascorbic acid (vitamin C) and carotenoids (total carotenoids reaching 5.58 mg/100 g) were reported as part of its chemical composition, linking them to health-promoting properties [8].

### 2.2. Specialised Metabolites

The secondary metabolites profile of *Sonchus asper* is characterised by a diverse array of bioactive compounds, including phenolic acids, flavonoids, coumarins, terpenoids, and sesquiterpene lactones, many of which have been structurally elucidated.

Nevertheless, phenolic acids constitute a major class of secondary metabolites present in the polar extracts of *S. asper*. Identified components from this group include caffeic acid, chlorogenic acid, rosmarinic acids, gallic acid and isochlorogenic acid, which are widely recognized for their antioxidant properties [5]. High total phenolic content has been reported, particularly in polar extracts, indicating their central role in free radical scavenging activity [7]. More recent metabolomic data additionally indicate the presence of chicoric acid, caftaric acid, and 3,4-dihydroxycinnamic acid derivatives. Also, small organic acids, like tartaric and malic acids, were detected in its extracts [5]. These compounds are key contributors to antioxidant capacity and are typical hydroxycinnamic acid derivatives in Asteraceae. Also, other phenolic derivatives were listed, like roseoside and dihydroroseoside, that contribute to the plant’s biological activity, including antioxidant and anti-inflammatory effects, and are typical of species within the Asteraceae family [6].

Flavonoids are among the most abundant and biologically active constituents of *S. asper*. The identified molecules from this group are, among others, luteolin, luteolin glucuronide and luteolin-7-glucoside, apigenin, apigenin-3-glucoside and apigenin glucuronide, catechin, rhamnetin, isorhamnetin or quercetin and its derivatives (quercetin-3-glucoside, quercetin-3-galactoside) [5,6,7].

These flavonoids exhibit strong antioxidant, anti-inflammatory, and cardioprotective activities, largely due to their polyhydroxylated structures [9]. Coumarins identified in *Sonchus asper* include aesculetin and cichoriin, which serve both as chemotaxonomic markers and bioactive compounds, followed by esculin [6,7]. In the end, the occurrence of terpenes, and particularly melampolide-type compounds as specialised metabolites in *S. asper* extracts was also noted. These include: 11β,13-dihydrourospermal A, 15-*O*-β-D-glucopyranosyl-11β,13-dihydrourospermal A, 15-*O*-β-D-glucopyranosylurospermal A, 15-*O*-[6′-(p-hydroxyphenylacetyl)]-β-D-glucopyranosylurospermal A, and 14-*O*-methylacetal-15-*O*-[6′-(p-hydroxyphenylacetyl)]-β-D-glucopyranosylurospermal A [10]. The aforementioned natural products often occur as glycosides and are characteristic of the Asteraceae botanical family, contributing to diverse biological activities, including anti-inflammatory and cytotoxic effects.

As proven above, the phytochemical profile of *Sonchus asper* demonstrates a particularly rich and chemically diverse composition, encompassing both primary metabolites (e.g., essential amino acids such as lysine, leucine, and tryptophan; polyunsaturated fatty acids including linoleic and α-linolenic acids; as well as vitamins and carotenoids) and a wide spectrum of secondary metabolites, notably flavonoids (luteolin, apigenin, quercetin derivatives), phenolic acids (chlorogenic, caffeic, chicoric acids), coumarins (aesculetin, cichoriin), and sesquiterpene lactones of the urospermal A type. Such a broad metabolite spectrum indicates a high potential for multilayer biological activity, including antioxidant, anti-inflammatory, and cytoprotective effects. The coexistence of these bioactive compounds, particularly polyphenols and unsaturated fatty acids, suggests that *S. asper* may represent a valuable source of functional ingredients for cosmetic applications, where modulation of oxidative stress, skin barrier support, and anti-ageing activity are of primary importance.

**Figure 1:**
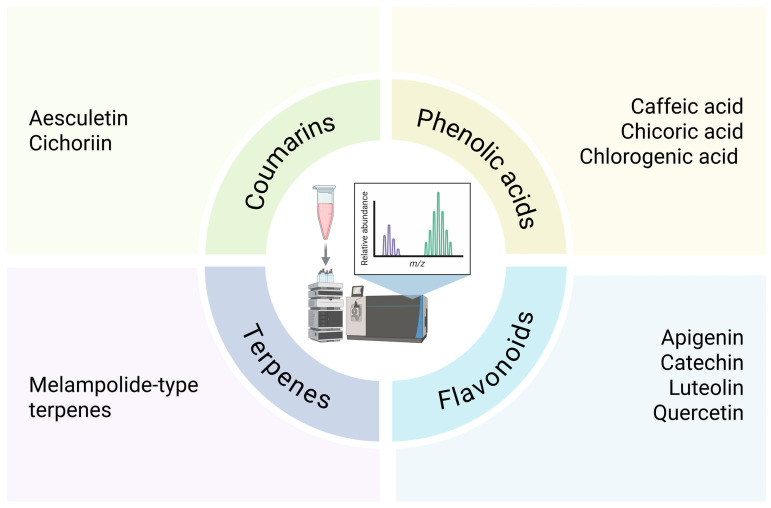
The secondary metabolites profile of *Sonchus asper*. Created in BioRender. Wawruszak, A. (2026) https://BioRender.com/4n391v2 (accessed on 24 May 2026).

From a cosmetic and industrial perspective, the standardisation of *S. asper*-derived raw materials is important to ensure consistent quality and reproducibility between batches. Based on current phytochemical data, a limited set of well-characterised compounds can be considered useful as potential marker compounds for quality control in cosmetic applications. These include key phenolic acids such as chlorogenic acid and caffeic acid, flavonoid glycosides including luteolin-7-*O*-glucoside and apigenin-7-*O*-glucoside as well as selected quercetin derivatives. In addition, representative fatty acids such as linoleic acid and α-linolenic acid may be useful for characterising the lipid fraction of the extract. These compounds are consistently reported in *S. asper* and are linked to its antioxidant, anti-inflammatory, and antimicrobial properties. However, their suitability as standardisation markers may depend on factors such as extraction method, plant part, and analytical approach. For this reason, chromatographic techniques such as HPLC and LC–MS/MS) are recommended for their reliable identification and quantification in quality control workflows. The proposed marker compounds for standardisation of *S. asper*-derived cosmetic raw materials are summarised in Figure 1 and in Supplementary Table S1.

## 3. Cosmetic Properties of *Sonchus asper*

**Figure 2:**
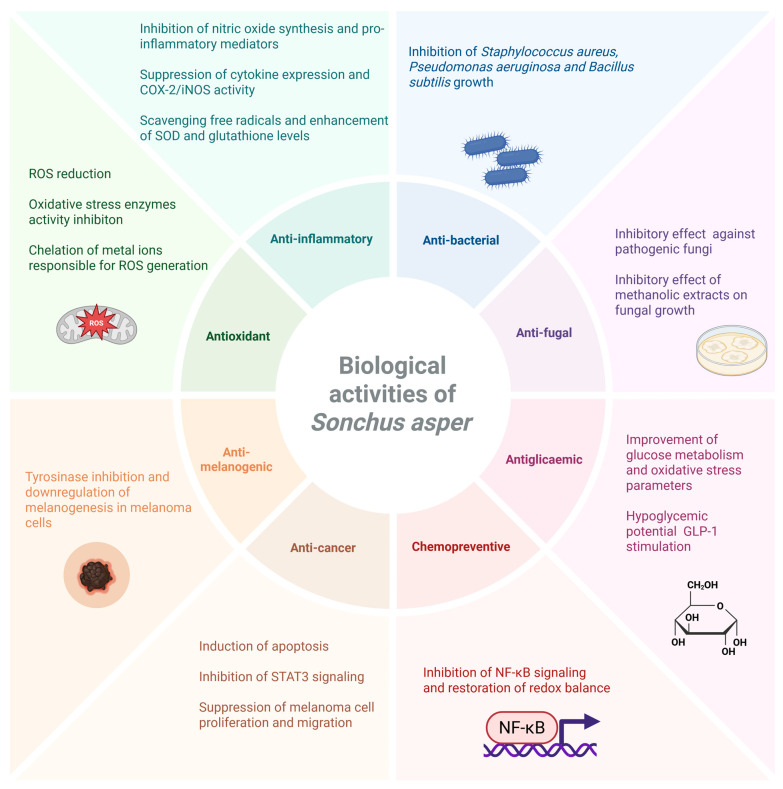
The cosmetic properties of *Sonchus asper*. Created in BioRender. Wawruszak, A. (2026) https://BioRender.com/uhpojqv (accessed on 24 May 2026).

Available literature indicates that *Sonchus asper* exhibits a broad spectrum of biological activities, including antioxidant, anti-inflammatory, antimicrobial, and metabolic effects, which may be of relevance for its potential application in cosmetic formulations (see Figure 2).

The current evidence is derived from diverse experimental approaches and, in some cases, remains in its early stages of investigation. A structured overview of the applied methodologies and reported biological endpoints is provided in the Table S2 in the Supplementary Material. Importantly, many of the primary and secondary metabolites identified in *Sonchus asper* correspond to compounds already listed in the CosIng database, where they are assigned defined cosmetic functions. This highlights that the phytochemical profile of *S. asper* includes ingredients with established roles in cosmetic formulations, such as antioxidants, emollients, and skin conditioning agents. A summary of selected compounds, along with their corresponding CosIng functions, is presented in Table 1.

### 3.1. Antioxidant Activity

As a member of the Asteraceae family, *Sonchus asper* is rich in antioxidant compounds, making it a valuable resource in medicine and pharmacy. It has been demonstrated that *S. asper* plant extracts contain ascorbic acid and a variety of polyphenolic compounds, particularly flavonoids and phenolic acids, which are attributed with strong free radical scavenging activity [5,6,9,11,12]. The results of the antioxidant activity assessment are presented further and in Table 1 and Table 2.

Phenolic compounds exhibit a strong capacity to scavenge reactive oxygen species (ROS), inhibit enzymes involved in oxidative stress, and chelate metal ions responsible for ROS generation. ROS are generated via the partial reduction of oxygen during lipid peroxidation, yielding oxygen-centred radicals, including HO•, O_2_•^−^, RO•, NO•, and ROO•. In addition, non-radical species such as H_2_O_2_ and O_3_ may be converted into reactive radicals and/or act directly as oxidising agents [13].

Oxidative stress is defined as an imbalance between oxidants and antioxidants, resulting in disturbances in cell proliferation and apoptosis. It also affects key cellular control mechanisms, including membrane permeability and the activity of antioxidant and enzymatic systems, ultimately disrupting replication, transcription, and translation processes. The superoxide anion (O_2_•^−^) is the primary ROS and is converted into H_2_O_2_ and subsequently into the highly reactive hydroxyl radical (OH•) [14]. Although endogenous defence systems, including antioxidant enzymes and low-molecular-weight compounds, are present, they are often insufficient to fully prevent oxidative damage, thereby requiring additional support from exogenous sources.

The antioxidant activity of *S. asper* extracts has been evaluated using various analytical methods, most commonly the Folin–Ciocalteu assay, ABTS (2,2′-azino-bis(3-ethylbenzothiazoline-6-sulfonic acid) assay, DPPH radical-scavenging assay (2,2-diphenyl-1-picryl-hydrazyl), ferric reducing antioxidant power (FRAP), and cupric reducing antioxidant capacity (CUPRAC) assays [1,2,4,6]. The Folin–Ciocalteu method is a reference assay for determining total phenolic content (TPC). It is based on the oxidation of phenolic compounds by a phosphomolybdic–phosphotungstic acid complex in the presence of gallic acid as a standard, resulting in the formation of a blue-colored product. Absorbance is measured at λ = 765 nm and is directly proportional to TPC [13,15].

The non-specific DPPH assay is widely used to determine antioxidant activity. The DPPH radical exhibits a violet colour in solution with maximum absorbance at λ = 515 nm. Upon reaction with radical scavengers, it accepts electrons, leading to a colour change from violet to yellow. Results are expressed either as the percentage of radical scavenging or as the IC_50_ value, which indicates the concentration required to neutralise 50% of the radicals. The ABTS assay measures the ability of antioxidants to neutralise the ABTS•^+^ radical cation, which exhibits a blue-green colour with maximum absorbance at λ = 734 nm. A decrease in absorbance corresponds to increased antioxidant activity [15]. 

The CUPRAC assay is based on spectrophotometric measurement of a coloured complex formed by reaction of Cu(I) ions with bathocuproine or neocuproine. Similarly, the FRAP assay measures the absorbance of a coloured complex formed by the reaction between TPTZ (2,4,6-tripyridyl-s-triazine) and antioxidants [16].

Scientific literature provides numerous examples of studies confirming the strong antioxidant potential of *S. asper* extracts. Parisi et al. investigated the antioxidant activity of extracts obtained from raw and cooked edible leaves of *S. asper*, prepared using ultrasound-assisted (UAE) and microwave-assisted (MAE) extraction, as well as extracts from discarded leaves (SAD) and their liposomal formulations coated with Eudragit. Cooked leaf extracts were prepared by boiling plant material (20 g) for 2 min, followed by ultrasonic (320 W) or microwave (up to 1000 W) treatment. Extraction was performed for 15 min using an EtOH:H_2_O (7:3) solvent system at a 1:10 ratio. Further, liposomes were prepared using Phospholipon 90G, stearylamine, and SAD extract, dispersed in phosphate buffer, sonicated, and subsequently coated with 0.1% (*w*/*v*) Eudragit to enhance gastrointestinal stability and bioavailability [6].

Another study demonstrates the results of the TPC determination using the Folin–Ciocalteu method, while the antioxidant activity was assessed using DPPH and FRAP assays in STC-1 enteroendocrine intestinal cells. The highest phenolic content and antioxidant potential were observed in Eudragit-coated liposomes (79.17 ± 6.03 mg GAE/g), whereas the lowest values were recorded for raw leaf extracts obtained via ultrasound (20.08 ± 3.54 mg GAE/g). Corresponding DPPH values were 266.58 ± 23.53 mg TE/g and 15.67 ± 1.96 mg TE/g, respectively, while FRAP values were 2526.34 ± 151.25 mg TE/g and 9.7 ± 0.28 mg TE/g, respectively. Notably, cooked leaf extracts exhibited higher levels of bioactive metabolites than dry material, likely due to thermal disruption of cellular structures, thereby facilitating compound release. Additionally, discarded leaf extracts demonstrated particularly high antioxidant activity [6].

Altin et al. evaluated the antioxidant activity and flavonoid content in components of çalkama, a traditional Turkish dish composed of wild edible greens, including *S. asper*. Extracts of flavan-3-ols and flavones/flavonols/flavanones were analyzed for total phenolic content (TPC), total flavonoid content (TFC), and total antioxidant capacity (TAC) using the CUPRAC assay. For *S. asper*, flavan-3-ol extracts yielded: TPC = 56.54 ± 3.86 mg GAE/g dry weight, TFC = 13.16 ± 1.69 mg CA/g, TAC = 216.94 ± 3.30 mg TE/g. Flavone/flavonol/flavanone extracts showed: TPC = 12.49 ± 1.72 mg GAE/g, TFC = 19.37 ± 1.57 mg CA/g, TAC = 728.96 ± 15.30 mg TE/g. The aforementioned values were determined by the presence of quercetin, rhamnetin, and isorhamnetin, while apigenin was the predominant flavone in the tested samples. Also, among key phenolic acids, chlorogenic, gallic, and rosmarinic acids were identified [12].

Another study compared the antioxidant activity of hydroethanolic extracts from raw and cooked *S. asper* leaves. The described results indicated that raw leaf extracts exhibited higher antioxidant potential, attributed to a greater phenolic content, which is partially degraded during cooking. As a result, the raw extracts exhibited 2.2-fold greater inhibition of lipid peroxidation and 1.8-fold greater inhibition of oxidative hemolysis than the decoctions [11].

The radical scavenging properties of *S. asper* decoctions were characterised with an IC_50_ value of 13.56 ± 3.19 µg/mL in the DPPH assay related to an abundant presence of polyphenols [5]. On the other hand Khan et al. demonstrated that the methanolic extracts from the plant contained the highest levels of phenolics (332 ± 1.53 mg GAE/g) and flavonoids, as well as the strongest antioxidant activity across multiple assays (DPPH, ABTS, ROS scavenging, and metal chelation), compared to other solvent fractions [9]. The results obtained by de Paula Filho et al. were in line with the formerly described and stood for the highest efficiency of antiradical properties in methanolic extracts. According to the authors, these samples had the highest phenolic content, while acetone extracts contained the highest flavonoid levels. Moreover, the antioxidant activity measured in ABTS, FRAP, and DPPH assays confirmed the plant’s strong activity, with methanolic extracts showing superior reducing power [8]. The summary of in vitro antioxidant activity of *S. asper* is presented in the Table 2 and Table 3.

Current evidence unequivocally positions *Sonchus asper* as a potent source of natural antioxidants. The overwhelming majority of studies consistently report high antioxidant capacity, demonstrated across multiple in vitro platforms, including free radical scavenging assays (DPPH, ABTS), reducing power methods (FRAP, CUPRAC), and quantitative determinations of total phenolic and flavonoid content (TPC, TFC), as well as complementary assays such as metal chelation and lipid peroxidation inhibition. These convergent findings highlight a strong correlation between antioxidant efficacy and the abundance of polyphenolic constituents, particularly flavonoids and phenolic acids. Nevertheless, it must be underscored that these results are largely derived from in vitro spectrophotometric models, which do not fully capture the complexity of biological systems; thus, expanded in vivo investigations or cell-based assays are critically needed to validate bioavailability, efficacy, and safety profiles.

From a dermocosmetic standpoint, the antioxidant activity of *S. asper* is highly relevant. Oxidative stress is a central driver of skin ageing and inflammation, contributing to collagen degradation, elastin damage, and barrier dysfunction. Antioxidants mitigate these processes by neutralising reactive oxygen species and modulating redox-sensitive signalling pathways, thereby supporting skin integrity and delaying photoaging [17]. Moreover, antioxidant compounds play a dual technological role by enhancing the oxidative stability of cosmetic formulations, limiting degradation of lipids and active ingredients, and ultimately extending product shelf life [18].

The antioxidant activity of *S. asper* can also be placed in the context of other well-characterized plant-derived antioxidants widely used in cosmetic and pharmaceutical applications [19]. For instance, extracts of *Vitis vinifera* rich in phenolic acids, flavonols, and stilbenoids demonstrated moderate DPPH radical scavenging activity (up to 33.57% inhibition), compared to 73.73% for the Trolox reference, and metal chelating activity of up to 50.93% versus 97.14% for EDTA [20]. In another study, a phenolic-rich extract of *V. vinifera* exhibited an IC_50_ of 23.1 μg/mL and an antioxidant activity index (AAI) of 1.02, comparable to those of vitamin C and vitamin E, indicating strong free-radical scavenging capacity [21]. Similarly, *Rosmarinus officinalis*, a well-established cosmetic antioxidant, showed high radical scavenging activity, with DPPH and ABTS inhibition reaching approximately 79% and 70–79%, respectively, depending on the chemotype [22]. Extracts of *Camellia sinensis*, widely regarded as a benchmark botanical antioxidant, demonstrated EC_50_ values of approximately 24.3 μg/mL, while at concentrations of 100–200 μg/mL, DPPH scavenging activity exceeded 95%, surpassing that of L-ascorbic acid [23].

In comparison, the antioxidant activity of *S. asper*, as demonstrated in multiple in vitro assays (DPPH, ABTS, FRAP, CUPRAC), falls within a range consistent with that of these well-known plant extracts, particularly given differences in extraction methods and experimental conditions. The relatively low IC_50_ values reported for certain *S. asper* extracts, together with high total phenolic content and strong reducing capacity, indicate that its antioxidant performance is comparable to other phenolic-rich botanical sources. These findings support the classification of *S. asper* as a promising and underexplored source of natural antioxidants with potential applications in cosmetic formulations.

### 3.2. Anti-Inflammatory Activity

Anti-inflammatory activity is a key attribute in cosmetic applications, as cutaneous inflammation underlies numerous skin concerns, including irritation, erythema, premature ageing, and barrier dysfunction. The ability to modulate inflammatory pathways is therefore essential for maintaining skin homeostasis and improving both the efficacy and tolerability of cosmetic formulations [24]. 

*Sonchus asper* is widely used in traditional medicine as an anti-inflammatory and analgesic agent; leaf extracts are commonly used to treat wounds and boils. These effects are primarily attributed to the presence of flavonoids—polyphenolic compounds with well-documented antioxidant, anti-inflammatory, and analgesic activities, including free radical scavenging and inhibition of hydrolytic and oxidative enzymes [25,26]. Tripathi et al. [27] highlighted the anti-inflammatory potential of *S. asper* in their review on this invasive species in India. Additionally, Zarei et al. evaluated the anti-inflammatory and antinociceptive effects of *S. asper* leaf extract and its major constituent, apigenin-7-glucoside (Ap7G), in male mice [28]. Ap7G, a flavonoid with additional anticancer potential [29], demonstrated significant biological activity. Under experimental conditions, both the hydroalcoholic extract and Ap7G exhibited pronounced antinociceptive and anti-inflammatory effects [28]. These were assessed using standard nociceptive models, including writhing, tail-flick, and formalin- and glutamate-induced paw-licking tests, as well as the xylene-induced ear oedema model for inflammation. Ap7G showed significant activity across all assays. The authors suggested that these effects may involve modulation of glutamatergic pathways via opioid receptor interactions and suppression of inflammatory mediators. The observed activity was attributed to the presence of bioactive compounds, including flavonoids and terpenoids (mono-, sesqui-, and diterpenes). In particular, inhibition of nitric oxide (NO) synthesis was proposed as a key mechanism, consistent with earlier findings by Shimizu et al. [30], who demonstrated reduced NO production mediated by sesquiterpenoids.

Wang et al. investigated the anti-inflammatory activity of an ethyl acetate extract from the aerial parts of *S. asper* in lipopolysaccharide (LPS)-stimulated RAW264.7 macrophages. The extract (100 µg/mL) almost completely inhibited nitric oxide production. Furthermore, concentrations ≥ 25 µg/mL significantly suppressed the expression of pro-inflammatory mediators, including iNOS, COX-2, IL-1β, IL-6, and TNF-α. The extract also enhanced superoxide dismutase (SOD) activity and intracellular glutathione (GSH) levels, indicating improved redox balance. Given the central role of SOD and GSH in maintaining intracellular oxidative homeostasis, these findings suggest that the anti-inflammatory effects of *S. asper* are closely associated with its antioxidant properties and the attenuation of oxidative stress in activated macrophages [31]. This particular activity is strictly related to the composition of the extracts. As described above, the presence of rutin, caffeic acid, and quercetin as major constituents of the plant determines the total activity of the plant extracts. These compounds are well known for their biological activity: rutin exhibits strong anti-inflammatory effects, while caffeic acid acts as an effective antioxidant that scavenges reactive species, including singlet oxygen, thereby reducing lipid peroxidation [32,33]. In this context, *Sonchus asper* is particularly noteworthy, as its extracts exhibit pronounced anti-inflammatory effects that distinguish this species from many other plant-derived ingredients.

### 3.3. Antimicrobial and Antifungal Properties of the Plant

Antimicrobial activity is an important feature of plant-derived compounds in cosmetic applications. It contributes both to product preservation by limiting microbial contamination and to the maintenance of skin microbiota balance. This dual functionality is particularly relevant in the context of acne, irritation, and other microbiologically driven skin disorders. In addition, natural antimicrobial agents are increasingly explored as alternatives to synthetic preservatives, aligning with current trends in cosmetic formulation.

#### 3.3.1. Inhibition of *Staphylococcus aureus*

*Staphylococcus aureus* is a Gram-positive bacterium commonly associated with skin infections, including acne, abscesses, and recurrent dermal inflammations. Its increasing resistance to antibiotics, particularly in methicillin-resistant strains (MRSA), highlights the need for alternative antimicrobial agents [34].

The antibacterial activity of *S. asper* against *S. aureus* has been demonstrated in experimental studies using different extraction solvents [35]. Among these, methanolic extracts of aerial plant parts are most frequently reported as the most effective, exhibiting a concentration-dependent inhibitory effect. Notably, Xia et al. [36] reported particularly low minimum inhibitory concentration (MIC) values for the ethyl acetate fraction (0.039 mg/mL) and the n-butanol fraction (0.078 mg/mL), indicating strong antibacterial potency. These results stand in contrast to other studies using different solvents; for instance, Mallik et al. [37] reported an MIC of 0.8 mg/mL for methanol extracts, whereas Jimoh et al. [38] reported significantly higher values of 2.0 mg/mL for acetone and aqueous extracts. The latter authors reported higher inhibitory concentrations against *Staphylococcus aureus*, namely 2.0 mg/mL for the acetone extract and 2.0 mg/mL for the aqueous extract, respectively. Interestingly, the authors did not report the antibacterial activity for methanol extracts, although methanol extracts achieved the strongest antibacterial activity in most experiments. The available data suggest that *S. asper* exhibits promising antibacterial activity against *S. aureus*, with potential relevance for the management of acne and secondary skin infections.

#### 3.3.2. Inhibition of *Pseudomonas aeruginosa*

*Pseudomonas aeruginosa* is a Gram-negative opportunistic pathogen known for its high resistance to environmental stress and antimicrobial agents. It is frequently associated with wound infections, burns, and post-surgical complications, and represents a critical challenge in cosmetic microbiological safety assessments [39,40,41]. Studies indicate that *S. asper* extracts exhibit moderate antibacterial activity against *P. aeruginosa*. According to Khan et al., the methanolic fraction exhibited an MIC of 5 mg/mL [35,42]. These findings are further supported by R. Kausar et al., who also identified the methanolic extract as the most potent, with significant inhibitory effects observed at 15 mg/mL. In their study, ethanol extracts showed moderate activity, while aqueous extracts remained the least effective. This hierarchical efficacy (methanol > ethanol > water) suggests that the bioactive compounds responsible for the anti-pseudomonal activity of *S. asper* are primarily medium- to high-polarity.

#### 3.3.3. Inhibition of *Bacillus subtilis*

*Bacillus subtilis* is a Gram-positive bacterium commonly found in the environment and occasionally associated with opportunistic infections, particularly in immunocompromised individuals. It includes three subspecies, subtilis, spizizenii and inaquosorum that are also relevant in the context of cosmetic contamination. *B. subtilis* is generally considered a nonpathogenic organism and is even used in probiotics and the pharmaceutical industry [43,44,45]. *B. subtilis* can cause folliculitis and wound infection and can colonise surgical or traumatic wounds, leading to local infection. Available data on the activity of *S. asper* against *B. subtilis* are limited. Nevertheless, Khan et al. [42] observed the antibacterial activity against *B. subtilis* of ethyl acetate and methanol extracts of *S. asper* at a concentration of 1 mg/mL. R. Kausar et al. [35] also showed methanol extract to be most effective against the bacterium *B. spizizinii* which is closely related to *B. subtilis*. Given that *B. subtilis* is a common contaminant in cosmetic formulations, the demonstrated inhibitory effects (at 1 mg/mL) suggest that *S. asper* extracts could serve as natural preservative boosters. However, further research is required to evaluate these effects in complex cosmetic matrices to ensure long-term microbiological stability.

#### 3.3.4. Antifungal Activity

Preliminary studies suggest that *S. asper* extracts may also exhibit antifungal activity against selected pathogenic fungi: *Aspergillus niger*, *Fusarium solani*, *Aspergillus flavus*, *Aspergillus fumigates*, *Rhizoctonia solani*, *Candida albicans* and *Botrytis cinerea*. Many of them are important in fungal infections of the skin and nails, known in medicine as mycoses, which are growing in resistance and adaptability of fungi [46]. Additionally, they can easily infect healthy people. Similarly to the activity against bacteria, the most significant effect was observed with the methanolic extracts of *S. asper*. 

Khan et al. examined the antifungal activity at a concentration of 24 mg/mL of *S. asper* against 4 different strains: *A. niger*, *F. solani*, *A. flavus* and *A. fumigatus*. Studies have shown that all tested extracts exhibit antifungal properties, though with varying degrees of effectiveness. The methanolic extract proved most effective, inhibiting the growth of *A. niger* and *A. fumigatus* by 80% and 85%, respectively. Butanolic extract was effective only against the *Fusarium solani* strain at a high level of over 90%. None of the extracts were highly effective against the *Aspergillus flavus* strain [42]. 

Upadhyay et al. demonstrated the activity of *S. asper* against two fungal species: *C. albicans* and *A. flavus*. The authors did not observe antifungal activity for any of the extracts tested. The authors attribute this resistance to the complex chitinous structure of the fungal cell wall [47]. The antifungal activity against *R. solani* was also examined by Rafiq M. et al. They studied the activity of methanol extract from the root of *S. asper* and they obtained results indicating antibacterial activity for different concentrations of 1.56–200 mg/mL, respectively 57–97% [48]. 

Promising results were obtained by Ejaz T. et al. who tested the extracts of the above-ground parts of *S. asper* against the strains of *B. cinerea* and *R. solani*. The methanolic extract from the aerial parts showed antifungal activity against *R. solani*, but weaker compared to *B. cinerea*. However, *R. solani* and *B. cinerea* do not significantly affect human skin and do not contribute to skin infections [49].

Bioactive compounds from *S. asper* may be effective in combating various strains of bacteria responsible for skin infections, which may be particularly important in the context of skin inflammation and infections. The results indicate that the methanol extract has the strongest effect on both bacterial and fungal infections. The biological activity of such extracts is definitely higher compared to the use of other solvents. The activity of other extracts against individual pathogens should also be investigated, e.g., butanolic extract was effective against the *F. solani*, but there is not enough research to compare it with methanol extracts. Analyzed studies suggest that *S. asper* has antibacterial and antifungal properties and is a promising subject for further research in the context of natural antimicrobials for use in cosmetics and dermatology. Thanks to its antibacterial and antifungal properties against the most common pathogens causing bacterial infections and candidiasis of the skin such as *C. albicans* and *S. aureus*, the leaves or the entire *S. asper* plant can be used in the treatment of skin infections, e.g., antibacterial and antifungal creams or ointments and in preparations supporting wound healing or medicated nail polishes. It is also possible to use it in natural plant cosmetics for skin care, but it is necessary to examine the safety of this plant in more detail. Additionally, activity against *S. aureus* was high but not against *C. albicans*, which is not promising because *C. albicans* is one of the fungal species responsible for frequent and recurrent skin infections. However, the activity of methanol extracts against the fungi *A. niger* and *A. fumigatus* was very high, and these pathogens are capable of causing cutaneous mycoses. It is possible that using other extraction methods would make it more effective against candida species. The origin of the plant and the content of active compounds may also be an important aspect in the effectiveness of *S. asper* and may explain the different concentrations obtained in different publications. *S. asper* has antibacterial potential, especially against Gram-positive bacteria and, to a lesser extent, inhibits fungal pathogens (see also Table 4).

Overall, the presented data indicate that *Sonchus asper* exhibits measurable antimicrobial activity, particularly against Gram-positive bacteria such as Staphylococcus aureus, which represents a clear strength of the reviewed evidence. The relatively low MIC values reported in some studies, together with consistent activity of methanolic extracts, support the presence of bioactive compounds with genuine antibacterial potential. This is of particular relevance in cosmetics, where activity against *S. aureus* may contribute to managing acne-related microbiota imbalance and improving product preservation.

However, the evidence base also shows notable limitations. The reported antimicrobial effects are highly variable and strongly dependent on extraction method, solvent, and plant material, which reduces comparability between studies. In addition, most data are derived from in vitro assays using crude extracts, with limited standardisation and a lack of mechanistic insight. Activity against Gram-negative bacteria such as *Pseudomonas aeruginosa* is considerably weaker, and antifungal effects are inconsistent, particularly against clinically relevant species such as *Candida albicans*. These inconsistencies suggest that the antimicrobial spectrum of *S. asper* is relatively narrow and not yet fully characterised.

Importantly, there is a lack of studies evaluating the efficacy of *S. asper* extracts in complex cosmetic formulations or in vivo skin models, which limits the direct translation of these findings into practical applications. While the antimicrobial properties of *S. asper* are promising, especially against Gram-positive bacteria, they should currently be regarded as supportive rather than as standalone functional claims. Further research focusing on standardisation, active compound identification, formulation stability, and in vivo validation is required to assess its potential as a cosmetic antimicrobial agent fully.

### 3.4. Antiglycaemic Effects of S. asper

Diabetes is a global health problem associated with high morbidity and mortality across diverse populations [50]. Its pathogenesis is primarily linked to impaired glucose homeostasis, including reduced insulin secretion, decreased insulin sensitivity, and increased intestinal glucose absorption. These disturbances lead to chronic hyperglycaemia, which contributes to oxidative stress, lipid metabolism disorders, and progressive tissue damage. Natural products represent an important source of antidiabetic agents [51]. Their effects are attributed to bioactive compounds such as flavonoids, alkaloids, terpenoids, and glycosides, which act through multiple mechanisms, including stimulation of insulin secretion, inhibition of glucose absorption, modulation of glucose transporters, and improvement of insulin sensitivity [52]. In particular, flavonoids have been shown to inhibit carbohydrate-digesting enzymes such as α-glucosidase, thereby delaying glucose absorption and reducing postprandial glycaemia [51]. Studies on *Sonchus asper* further support the antidiabetic potential of plant-derived compounds. In vitro experiments demonstrated that *S. asper* extracts are non-cytotoxic and capable of modulating glucose-related pathways, including stimulation of GLP-1 secretion, a key regulator of insulin release and glucose metabolism [53,54,55]. Administration of methanolic extracts in streptozotocin-induced diabetic rats resulted in reduced blood glucose levels, improved antioxidant enzyme activity, decreased lipid peroxidation (TBARS), and improved lipid profiles [56]. These findings are supported by further studies demonstrating dose-dependent hypoglycaemic effects of *S. asper* extracts in alloxan-induced diabetic models, with efficacy comparable to the standard antidiabetic drug glipizide [57].

Beyond direct glucose regulation, chronic hyperglycemia is closely associated with increased oxidative stress, inflammation, and the formation of advanced glycation end products (AGEs). These mechanisms are of interest not only in metabolic regulation but also in preventing glycation-related skin damage [56,57].

#### The Importance of *S. asper* Antidiabetic Properties in the Context of Skin Biology

Although most available studies focus on systemic antidiabetic effects following oral administration, the identified bioactive compounds may also be considered for topical applications. In this context, their antioxidant, anti-inflammatory, and antiglycation properties are particularly relevant for localised skin protection and cosmetic use. The antidiabetic properties of plants can affect the skin on three levels: enzymatic, cellular, and biochemical [58,59,60].

At the biochemical level, persistently high glucose levels lead to glycation, in which sugar molecules non-enzymatically bind to the structural proteins of the skin, collagen and elastin. This reaction results in the formation of advanced glycation end products (AGEs), which destroy collagen cross-links, disrupt collagen structure by creating abnormal cross-links, alter tissue mechanical properties, and lead to loss of elasticity and wrinkle formation [61]. Phenolic compounds contained in *S. asper* exhibit antioxidant properties, neutralising reactive oxygen species (ROS) and limiting the formation of AGEs [62]. Because many phenolic compounds have limited bioavailability after oral administration [63], their topical use in cosmetic formulations may increase their availability within the skin and potentially enhance anti-ageing effects. In this context, the hypoglycemic properties of *S. asper*, which protect tissues from damage during metabolic disorders, may also indirectly protect the skin by limiting glycation processes [64].

At the enzymatic level, elevated glucose and ROS levels lead to activation of inflammatory pathways, including through the interaction of AGEs with the RAGE receptor, which results in increased expression of extracellular matrix-degrading enzymes, such as matrix metalloproteinases (MMPs), including MMP-1 (collagenase) and MMP-2. Excessive activity of these enzymes leads to collagen degradation and accelerated skin ageing [65]. Excessive MMP and ROS activity can further damage the skin, increasing the risk of skin cancer [66]. Compounds present in *S. asper*, such as caffeic acid and chlorogenic acid, can inhibit MMP activity and affect glucose metabolism by improving insulin sensitivity and slowing sugar absorption [67]. Flavonoids and coumarins can additionally inhibit the activity of matrix-degrading enzymes, such as elastase and hyaluronidase [68]. Furthermore, coumarin accelerates the removal of toxic AGEs by improving microcirculation and stimulating angiogenesis [69]. Terpene compounds and sesquiterpene lactones calm inflammation [70], which may limit the secondary activation of degradative enzymes. Specific amino acids and fatty acids, particularly concentrated in the roots of plants from the Asteraceae family, are potent signalling products that may help modulate the skin’s inflammatory response [67] and help protect collagen in the skin.

At the cellular level, chronic hyperglycemia and oxidative stress lead to fibroblast and keratinocyte dysfunction, reducing their ability to proliferate and synthesise collagen, and accelerating cellular ageing [71]. Bioactive compounds present in *S. asper* can modulate cellular responses by reducing oxidative stress and influencing inflammation-related signalling pathways (e.g., NF-κB), thereby promoting the maintenance of normal skin cell function and supporting regenerative processes [72,73].

These responses are accelerated by hyperglycemia, oxidative stress, and chronic inflammation. Due to their antioxidant, anti-inflammatory, and antiglycation properties, *S. asper* extracts may be a promising ingredient in dermatological and cosmeceutical formulations aimed at slowing skin ageing and supporting the treatment of chronic inflammatory conditions such as acne, atopic dermatitis, and irritation.

### 3.5. The Potential of S. asper in the Treatment of Skin Cancer

*S. asper* is a plant with well-documented biological activity, as discussed in the previous sections. However, literature specifically highlighting its direct anticancer activity in skin-related diseases remains limited. Most available studies report indirect protective effects or focus primarily on other species within the same genus, particularly *S. oleraceus*. To date, no peer-reviewed in vitro or in vivo studies have unequivocally demonstrated direct cytotoxic or antitumor effects of *S. asper* extracts against melanoma or other skin cancer models. Nevertheless, based on the existing literature, it can be hypothesised that *S. asper* possesses biological properties relevant to skin disease prevention or adjunctive therapy, particularly through mechanisms associated with chemoprevention.

In an early study, Hata et al. demonstrated that extracts from *S. asper* induced differentiation in B162F2 murine melanoma cells [74]. The authors concluded that this differentiation process may contribute to cytostatic effects, suggesting that plant-derived agents with differentiation-inducing properties could play a role in melanoma chemoprevention rather than direct tumour eradication. In the same study, *S. oleraceus* was also investigated and yielded comparable results. The authors further reported that in Cichorioideae species, lupeol represents a major triterpene constituent and may contribute to the observed cytostatic activity, as previously demonstrated in *Taraxacum* sp.

As the body’s primary barrier, the skin is continuously exposed to environmental and endogenous stressors that generate reactive oxygen species (ROS). When antioxidant defences are overwhelmed, oxidative stress may initiate cellular senescence, chronic inflammation, and carcinogenesis, either through direct damage to proteins, DNA, and lipids, or through dysregulation of redox-sensitive signalling pathways, including MAPK, Nrf2, JAK/STAT, NF-κB, PI3K/AKT/mTOR, SIRT1/FOXO [75]. These pathways are critically involved in melanoma initiation, progression, and therapy resistance.

In vivo studies conducted in Sprague–Dawley rats demonstrated that methanolic extracts of *S. asper* provide significant protection against carbon tetrachloride (CCl_4_)-induced liver [76] and lung [77] injury. Oral administration at doses of 100–200 mg/kg significantly reduced serum activities of hepatic enzymes (LDH, AST, ALT), improved lipid profiles, and restored antioxidant defence enzymes, including catalase, superoxide dismutase (SOD), glutathione peroxidase (GPx), glutathione S-transferase (GST), and glutathione reductase (GR). Furthermore, the extracts reduced oxidative stress markers, including lipid peroxidation products, hydrogen peroxide, nitrite levels, DNA fragmentation, and γ-glutamyl transferase activity. Histopathological analyses confirmed substantial attenuation of tissue damage in both organs. Although these investigations did not assess tumor-related endpoints, the demonstrated restoration of redox homeostasis and suppression of inflammation represent mechanistic processes strongly implicated in carcinogenesis and chemoprevention.

These observations are further supported by research conducted by Wang et al. [31], which evaluated the effects of the ethyl acetate fraction of *S. asper* on oxidative stress and inflammatory responses in LPS-stimulated RAW macrophages. Using the Griess assay, the authors demonstrated that the extract at 100 µg/mL nearly completely inhibited nitric oxide (NO) production. In contrast, a concentration of 25 µg/mL reduced NO levels by more than 50%. RT-PCR analysis revealed marked downregulation of key pro-inflammatory mediators, including iNOS, IL-1β, IL-6, TNF-α, and COX-2. Additionally, the extract significantly attenuated intracellular ROS accumulation and prevented LPS-induced loss of mitochondrial membrane potential. Enhanced SOD activity and elevated intracellular glutathione (GSH) levels further confirmed *S. asper*’s antioxidant capacity. Given the established roles of oxidative stress and chronic inflammation in melanoma development and progression, these findings support the potential chemopreventive relevance of *S. asper*, though they do not constitute direct evidence of anticancer efficacy.

*Sonchus asper* is rich in phytochemicals, including flavonoids, phenolic acids, and sesquiterpene lactones, which are widely recognised for their antioxidant, anti-inflammatory, and anticancer properties [6,78]. Compounds such as quercetin, catechin, luteolin, and sesquiterpene lactones have been extensively investigated for their ability to inhibit tumour cell proliferation, induce apoptosis, modulate oncogenic signalling pathways, and protect against UV-induced skin damage [79,80]. However, most mechanistic evidence for these compounds derives from studies using isolated phytochemicals rather than whole *S. asper* extracts.

Quercetin, a prominent flavonoid identified in *S. asper*, has demonstrated pronounced anticancer effects in melanoma models. In A375 and A2058 melanoma cell lines, quercetin inhibited cell proliferation, induced apoptosis, and reduced migratory and invasive behaviour by suppressing STAT3 phosphorylation and nuclear translocation, thereby downregulating STAT3-dependent genes such as MMP-2, MMP-9, VEGF, and Mcl-1 [81]. Overexpression of constitutively active STAT3 partially reversed these effects. In a separate study, quercetin significantly reduced viability and proliferation of A375SM melanoma cells in a concentration-dependent manner, while exerting minimal effects on A375P cells [82]. The compound induced apoptosis, increased Bax and cleaved PARP expression, activated JNK, p38, and ERK1/2 signalling, and downregulated Bcl-2 expression. Additionally, wound-healing assays revealed significant suppression of melanoma cell migration at concentrations of 40 and 80 μM. These findings highlight the relevance of *S. asper* phytochemicals as mechanistic contributors and emphasise the need for studies using standardised plant extracts.

Melanin plays a crucial protective role against UV-induced DNA damage and genetically driven alterations associated with melanoma development. Tyrosinase, a key regulatory enzyme in melanogenesis, is therefore considered an important therapeutic and preventive target. Natural products have been shown to modulate melanin synthesis by regulating tyrosinase activity and melanogenic signalling pathways [82,83]. Notably, *S. oleraceus*, a closely related species, demonstrated significant anti-melanogenic activity. Treatment of α-MSH-stimulated B16F10 melanoma cells with *S. oleraceus* extract (200 μg/mL) resulted in a 51.11% reduction in melanin content, accompanied by decreased intracellular tyrosinase activity. Although no comparable studies have yet been conducted for *S. asper*, the phytochemical similarity between these species supports the hypothesis that *S. asper* may exhibit analogous biological activity [38].

Traditionally, *S. asper* has been used in various folk medicine systems, particularly for wound treatment and promoting tissue repair [84]. However, the available scientific literature remains largely descriptive and lacks experimental or clinical validation of these uses. Nevertheless, ethnobotanical knowledge has historically provided an important foundation for drug discovery, supporting the relevance of traditional applications as a starting point for hypothesis-driven pharmacological research.

No direct experimental studies have investigated the wound healing properties of *S. asper*. In contrast, several studies have evaluated *S. oleraceus*-based formulations in wound healing models. In one study, topical application of balms and ointments containing *S. oleraceus* extract (5–15%) in Wistar rats resulted in accelerated wound contraction and complete closure by day 10 at the highest concentration [85]. Histological evaluation revealed increased collagen deposition and a higher type I-to-type III collagen ratio. A separate study [86] demonstrated enhanced fibroplasia, granulation tissue formation, and reduced necrosis following treatment with *S. oleraceus* extract alone. These effects were attributed primarily to flavonoids and tannins, which are also abundant in *S. asper*. Given the shared phytochemical profile, these findings provide a rationale—but not proof—for investigating *S. asper* in experimental wound-healing and skin-cancer-adjacent models.

Despite growing evidence supporting the antioxidant and anti-inflammatory activity of *S. asper*, direct experimental validation of its anticancer effects in skin-related models remains notably limited. To the best of our knowledge, no dedicated in vitro or vivo studies have systematically evaluated standardised *S. asper* extracts in melanoma or non-melanoma skin cancer models, which constitutes a critical and clearly defined research gap. As a result, the currently available data should be regarded as largely hypothesis-generating rather than confirmatory.

Future research should therefore prioritise mechanistic investigations of *S. asper* extracts using relevant melanoma cell lines and advanced skin-related experimental systems. Particular emphasis should be placed on clearly distinguishing direct cytotoxic activity against cancer cells from indirect chemopreventive effects mediated through modulation of oxidative stress, chronic inflammation, and UV-induced damage, as these processes differ substantially in their biological relevance and translational implications [87,88]. In this context, the use of advanced experimental platforms, such as three-dimensional skin equivalents or co-culture models incorporating melanocytes and keratinocytes, may provide more physiologically relevant insights into the cutaneous activity of *S. asper* and its potential role in skin cancer prevention.

In parallel, comparative phytochemical profiling and bioactivity assessments involving closely related species, particularly *S. oleraceus*, are warranted to evaluate the extent to which biological effects observed within the genus may be transferable or species-specific [36,81]. Such studies should be complemented by rigorous extract standardisation strategies focusing on dominant bioactive constituents, as variability in phytochemical composition remains a major obstacle to reproducibility and translational progress in phytopharmacological research.

Overall, the existing literature positions *S. asper* as a promising yet insufficiently explored candidate for skin cancer chemoprevention. Addressing the outlined experimental and methodological gaps will be essential for substantiating its therapeutic relevance and for determining whether the observed antioxidant and anti-inflammatory properties can be effectively translated into targeted strategies for melanoma prevention or adjunctive treatment.

### 3.6. Skin Barrier Suport

An important aspect of protecting the epidermal barrier in modern cosmetic formulations is maintaining the integrity of the natural moisturising factor (NMF). This factor is a mixture of low-molecular-weight, water-soluble substances (amino acids, pyroglutamic acid, lactate, urea, inorganic ions, and sugars) located within the corneocytes of the *stratum corneum*. It is responsible for maintaining optimal epidermal hydration and skin elasticity, and for regulating desquamation of the skin [89]. To support the stratum corneum’s NMF, cosmetics and dermatological products increasingly incorporate plant extracts rich in active ingredients preferred in cosmetic formulations. Among them, *Sonchus* sp. extracts play a significant role due to their high concentration of polysaccharides, free amino acids, mucilages, and lipids. Polysaccharides extracted from *Sonchus* species, in particular *S. arvensis*, *S. asper*, and *S. oleraceus*, are complex carbohydrate macromolecules such as galacturonans (homogalacturonan, a branched polysaccharide rhamnogalacturonan II, and galacturonan I, composed of dimers of rhamnose, galactose, arabinose, and galacturonic acid) [90]. These pectic polysaccharides exhibit strong water-binding properties and viscoelasticity, and their ability to retain water may help maintain optimal skin hydration, strengthen the skin’s protective barrier, and protect against harmful external factors. Moreover, *S. asper* is a rich source of essential amino acids, including L-lysine, L-threonine, L-isoleucine, L-leucine, L-phenylalanine, and L-tryptophan. These amino acids, when delivered topically in a cosmetic formulation, can directly replenish the pool of natural amino acids in the *stratum corneum*, which are lost with age or by detergent exposure, thereby restoring the skin’s structural ability to bind water [91]. *S. asper* extract, containing flavonoids (luteolin, apigenin, quercetin) and phenolic acids (chlorogenic, caffeic, chicory), has a strong antioxidant effect and inhibits the synthesis of inflammatory mediators (such as iNOS, COX-2, IL-1β) [92,93,94]. This action profile suggests that *S. asper* extract has the potential to support physiological keratinisation processes, thereby indirectly conditioning the correct endogenous NMF synthesis pathway.

Based on the presence of many preferred chemical components in *Sonchus* extracts, it can be hypothesised that, when applied to the skin, these compounds will form a thin, hydrophilic protective film on its surface [95]. This layer will not only limit transepidermal water loss (TEWL) but, above all, protect the naturally occurring, highly soluble NMF components in the epidermis from premature leaching by external factors [96].

## 4. New Approach Methodologies (NAMs) as a Necessary Aim for Assessing the Safety and Permeability of *Sonchus asper*

NAMs encompass strategies based on computational methods (in silico), cell- or reconstructed tissues-based assays (in vitro 2D/3D), and explants (ex vivo). A review of the current scientific literature reveals that crude extracts of *Sonchus asper* and related species, such as *S. oleraceus*, have not been directly tested in validated, reconstructed human epidermis (3D) models or ex vivo porcine skin models. This indicates a research gap that must be filled before this plant can be incorporated into commercial cosmetic formulations. Nevertheless, the potential safety and efficacy of this raw material in the context of NAMs can be predicted from the literature on the individual active ingredients of *S. asper*. Because porcine skin is considered one of the best-characterised skin surrogate models of the human epidermal barrier (*stratum corneum* thickness, lipid distribution), it is the standard for diffusion studies in Franz cells. The main flavonoids identified in *S. asper*, including quercetin, apigenin, and luteolin, were studied in three-dimensional skin models. Chessa et al. evaluated local delivery of quercetin from a w/o microemulsion using porcine skin mounted with Franz cells. They examined both penetration and local safety—assessing erythema, epidermal thickening, and inflammatory cell infiltration as indicators of irritation. They also demonstrated a protective effect of the formulation against UVB-induced decreases in reduced glutathione levels and increases in skin protease activity [97]. The study by Szulc-Musioł et al. evaluated hydrogels containing quercetin on porcine skin ex vivo—after 24 h, methylcellulose-based formulations showed the highest retention in the skin, confirming the suitability of this model for evaluating the formulation of active ingredients [98]. A combination of micropunctures and lipid microparticles was also tested to improve quercetin penetration through porcine skin—quercetin, as an ingredient, demonstrates poor penetration without support, which is important when planning formulation studies [99]. Literature data for the key metabolites of *S. asper*—chlorogenic acid and caffeic acid—obtained in ex vivo porcine skin models using Franz cells indicate that these compounds can penetrate the stratum corneum and retain within the skin, with the degree of penetration depending on the formulation and physicochemical properties of the molecules [100,101]. These properties are important from the point of view of dermocosmetic applications of topical antioxidants. In turn, the phytosterols present in the plant (beta-sitosterol), due to their lipophilicity, can interact with the lipid structure of the *stratum corneum*, supporting the skin’s barrier function [102]. In summary, although the current scientific literature on the *Sonchus* genus focuses primarily on traditional in vitro tests, the future of this raw material lies in the full implementation of NAMs methodology. Mapping the phytochemical profile of *S. asper*, combined with tests on 3D models and ex vivo permeation kinetics on porcine skin, is a necessary step that will allow the cosmetics industry to fully and safely utilise its application potential.

## 5. Materials and Methods

This review was conducted using a comprehensive and systematic literature search strategy. Scientific databases, including PubMed/MEDLINE, Scopus, and ScienceDirect, were used to identify publications on *Sonchus asper* and its biological and cosmetic potential.

The search was performed using combinations of keywords, including “*Sonchus asper*”, “antioxidant activity”, “anti-inflammatory”, “antimicrobial”, “antidiabetic”, “cosmetic applications”, and “skin”. Boolean operators (AND, OR) were applied to refine the search and ensure the inclusion of relevant studies.

Only peer-reviewed articles published in English were considered. The inclusion criteria combined the studies investigating the chemical composition, biological activity, and potential applications of *S. asper*, with particular emphasis on properties relevant to cosmetic science. Both in vitro and in vivo studies were included, with priority given to research published in the 21st century. The collected data were critically analyzed to identify the main bioactive compounds, mechanisms of action, and potential applications of *S. asper* extracts in dermatology and cosmetic formulations. The review aims to provide a coherent and up-to-date overview of the current state of knowledge and to highlight directions for future research.

## 6. Conclusions and Future Perspectives

This review highlights the significant biological potential of *Sonchus asper* (L.) Hill and its reliance as a multifunctional plant source for cosmetic applications. Due to its diverse phytochemical composition that includes both primary and secondary metabolites, *S. asper* is able to exhibit a broad spectrum of biological activities, including antioxidant, anti-inflammatory, antibacterial, antifungal, antiglycaemic, anti-aging, and potential anticancer effects, particularly in the context of skin-related conditions. Additionally, its ability to modulate microbial balance suggests a role not only in inhibiting pathogenic microorganisms but also in supporting the physiological skin microbiota and its beneficial impact on the condition of the NMF supports its usage in cosmetics. These properties position *S. asper* as a promising candidate for incorporation into modern cosmetic and dermocosmetic formulations. Notably, despite its long-standing use as an edible plant in Mediterranean regions, its cosmetic potential remains relatively underexplored. Future research should therefore focus on validating these effects using advanced biological models, including reconstructed human skin systems, as well as on comprehensive dermatological safety assessment. Such approaches will be essential to bridge the gap between experimental observations and practical application in cosmetic formulations. The available literature provides a strong preliminary foundation; however, current evidence is mainly based on in vitro studies, which limit direct translation into practical applications.

Importantly, although the biological and cosmetic potential of *Sonchus asper* is well supported by current literature, its safety profile remains insufficiently characterised. This represents a relevant limitation for future cosmetic applications, as natural origin does not inherently guarantee safety in topical use. In fact, consumers often incorrectly perceive plant-derived ingredients as inherently safe, whereas in practice, their toxicological evaluation is a critical step prior to formulation. In the cosmetic and raw materials industry, the responsibility for confirming the safety of botanical ingredients lies with toxicologists, who assess potential risks such as irritation, sensitization, and dose-dependent effects. Therefore, dedicated toxicological and dermatological assessments will be required before its incorporation into cosmetic formulations, particularly with regard to irritation and sensitization potential.

Future research should therefore focus on in vivo studies and clinically relevant models to better elucidate the mechanisms of action, bioavailability, and safety profile of *S. asper* extracts and their individual constituents. Particular attention should be given to the development and optimization of formulation strategies and tests within complete cosmetic formulations using validated non-animal methodologies, such as reconstructed human skin or ocular models in accordance with OECD guidelines, to better reflect real application conditions and potential synergistic effects, including delivery systems that enhance the stability, skin penetration, and controlled release of active compounds. Such approaches are essential to determine which bioactive molecules effectively contribute to the observed cosmetic effects under real-use conditions. In the absence of ex vivo skin penetration studies (e.g., Franz diffusion cell models) and well-designed human clinical trials, the reported cosmetic potential should be regarded as preliminary and largely theoretical.

Furthermore, standardisation of extraction methods and phytochemical profiling is required to ensure reproducibility and consistency of results. Investigations into interactions between individual compounds may also provide valuable data on the total biological activity of the plant. From an industrial perspective, the sustainability, availability, and low-cost nature of *S. asper* further support its potential as an attractive raw material for the development of natural cosmetic products.

## Figures and Tables

**Table 1:** The identity and functions of the selected metabolites of *Sonchus asper* in the CosIng database.

Primary Metabolites of *Sonchus asper*	
Chemical compounds	Function according to CosIng
Ascorbic Acid	antioxidant, skin conditioning
Tocopherol (Vitamin E)	antioxidant, skin conditioning
Vitamin K	skin conditioning
Linoleic Acid	emollient, skin conditioning, surfactant
Alpha-Linolenic Acid	skin conditioning, emollient
L-Lysine	antistatic, hair conditioning, skin conditioning
L-Threonine	skin conditioning
L-Isoleucine	skin conditioning
L-Leucine	skin conditioning
L-Phenylalanine	skin conditioning
L-Tryptophan	skin conditioning
Secondary metabolites of *Sonchus asper*	
Phenolic compounds	Function according to CosIng
Caffeic Acid	antioxidant, masking
Chlorogenic Acid	antioxidant, skin conditioning
Rosmarinic Acid	antioxidant, skin conditioning
Gallic Acid	antioxidant, masking
Chicoric Acid	antioxidant, skin conditioning
Malic Acid	buffering, humectant, skin conditioning
Flavonoid compounds	
Quercetin	antioxidant, skin protecting
Quercetin-3-Glucoside	antioxidant
Quercetin-3-Galactoside	antioxidant
Luteolin	antioxidant, skin protecting
Luteolin-7-Glucoside	antioxidant
Apigenin	antioxidant, skin protecting
Catechin	antioxidant, skin conditioning
Isorhamnetin	antioxidant
Rhamnetin	antioxidant
Coumarin compounds	
Esculin	skin conditioning, tonic
Esculetin	antioxidant

**Table 2:** In vitro test results summarizing the antioxidant activity of various extracts *S. asper*.

			Type of Test						
Extract Type	Extraction Characteristics	Solvent	DPPH	FRAP	TBARS (EC_50_ μg/mL)	OxHLIA (IC_50_ μg/mL) After 60 min	OxHLIA (IC_50_ μg/mL) After 120 min	ABTS	Reference Compound/Literature
Raw U leaf extract	U (320 W, 15 min);	Hydroalcoholic	15.67 ± 1.96 mg TE/g	9.7 ± 0.28 mg TE/g	NR	NR	NR	NR	[6]
Cooked U leaf extract	2 min of cooking. U (320 W, 15 min)	Hydroalcoholic	19.47 ± 1.56 mg TE/g	16.34 ± 2.05 mg TE/g	NR	NR	NR	NR	
Raw MW leaf extract	MAE (1000 W, 5 min)	Hydroalcoholic	17.88 ± 1.87 mg TE/g	14.94 ± 1.85 mg TE/g	NR	NR	NR	NR	
Cooked MW leaf extract	2 min of cooking. MAE (1000 W, 5 min)	Hydroalcoholic	21.72 ± 2.49 mg TE/g	29.63 ± 2.14 mg TE/g	NR	NR	NR	NR	
SAD extract	U (320 W, 15 min); maceration	Hydroalcoholic	209.56 ± 19.14 (9.0 ± 0.94) mg TE/g	1540.77 ± 110.28 (12.33 ± 0.88) mg TE/g	NR	NR	NR	NR	
SAD eu-liposomes	U (320 W, 15 min); maceration; sonification	Hydroalcoholic	266.58 ± 23.53 (11.46 ± 1.1) mg TE/g	2526.34 ± 151.25 (20.35 ± 2.85) mg TE/g	NR	NR	NR	NR	
Hydroethanol raw leaf extract	Stirring for 1 h	EtOH: H_2_O (80:20)	NR	NR	144 ± 1 μg/mL	35 ± 3 μg/mL	112 ± 9 μg/mL	NR	Trolox/[11]
Decoction	Distillation for 5 min	Boiling water	NR	NR	281 ± 4 μg/mL	49 ± 4 μg/mL	116 ± 9 μg/mL	NR	
Hydroethanol raw leaf extract from decoction	Distillation for 5 min; stirring for 1 h	Boiling water EtOH:H_2_O (80:20)	NR	NR	318 ± 6 μg/mL	62 ± 2 μg/mL	130 ± 6 μg/mL	NR	
SAME	Maceration for 48 h at 25 °C;	Methanol	2.5 ± 0.05 μg/mL	64 ± 2.12 μg/mL	NR	NR	NR	53.4 ± 4.2 μg/mL	Ascorbic acid/[9]
SACE	Liquid–liquid partitioning for 6 h	Acetone	3.8 ± 0.2 μg/mL	87.8 ± 2.56 μg/mL	NR	NR	NR	74.2 ± 2.6 μg/mL	
SAEE	Liquid–liquid partitioning for 6 h	Ethanol	4.1 ± 0.32 μg/mL	100.4 ± 2.21 μg/mL	NR	NR	NR	83.4 ± 1.5 μg/mL	
SAHE	Liquid–liquid partitioning for 6 h	n-Hexane	12.2 ± 1.43 μg/mL	110.6 ± 1.67 μg/mL	NR	NR	NR	90.21 ± 2.8 μg/mL	
Ascorbic acid	-	Reference compound	3.61 ± 23 μg/mL	73.7 ± 3.4 μg/mL	NR	NR	NR	76.3 ± 2.15 μg/mL	
Leaf extract	Extraction in room temperature for 18–24 h	Acetone	85.6%	158.67 ± 21.89 μmol Fe (II)/g	NR	NR	NR	97.8%	BHT/[8]
Leaf extract	Extraction in room temperature for 18–24 h	Methanol	85.3%	298.56 ± 32.52 μmol Fe (II)/g	NR	NR	NR	98.0%	
Leaf extract	Extraction in room temperature for 18–24 h	Water	81.8%	18.32 ± 5.79 μmol Fe (II)/g	NR	NR	NR	99.1%	
Ascorbic acid	-	Reference compound	99.8%	1632.1 ± 16.95 μmol Fe (II)/g	NR	NR	NR	NR	
BHT	-	Reference compound	100%	63.46 ± 2.49 μmol Fe (II)/g	NR	NR	NR	99.3%	
Catechin	-	Reference compound	NR	972.02 ± 0.61 μmol Fe (II)/g	NR	NR	NR	NR	
Quercetin	-	Reference compound	NR	3107.29 ± 31.28 μmol Fe (II)/g	NR	NR	NR	NR	

ABTS—(2,2′-azino-bis(3-ethylbenzothiazoline-6-sulfonic acid) assay; BHT—butylhydroxytoluene; DPPH—2,2-diphenyl-1-picryl-hydrazyl assay; FRAP—ferric reducing antioxidant power assay; MW—microwaves; NR—not reported; OxHLIA—Oxidative Haemolysis Inhibition Assay; SAD—non-edible external hard leaves; eu—eudragit; SAME—methanol fraction; SACE—acetone fraction; SAEE—ethanol fraction; SAHE—n-hexane fraction; TE—Trolox; TBARS—Thiobarbituric acid reactive substances assay; U—ultrasound.

**Table 3:** Test results determining the content of substances with antioxidant activity in *S. asper* extracts.

Extract Type	Solvent	Type of Test			
		TPC	TFC	TAC	Literature
Raw U	Hydroalcoholic	20.08 ± 3.54 mg GAE/g	NR	NR	[6]
Cooked U	Hydroalcoholic	20.51 ± 1.18 mg GAE/g	NR	NR	
Raw MW	Hydroalcoholic	20.21 ± 2.47 mg GAE/g	NR	NR	
Cooked MW	Hydroalcoholic	43.85 ± 0.43 mg GAE/g	NR	NR	
SAD extract	Hydroalcoholic	63.36 ± 3.73 (1.69 ± 0.1) mg GAE/g	NR	NR	
SAD eu-liposomes	Hydroalcoholic	79.17 ± 6.03 (2.02 ± 0.02) mg GAE/g	NR	NR	
Flavonol-3-ol extracts	Methanol	56.54 ± 3.86 mg GAE/g	13.16 ± 1.69 mg CA/g s.m	216.94 ± 3.30 mg TE/g	[12]
Flavones/flavonols/flavanones	Methanol	12.49 ± 1.72 mg GAE/g	19.37 ± 1.57 mg CA/g s.m	728.96 ± 15.30 mg TE/g	
SAME	Methanol	332 ± 1.53 mg rutin/g	11.4 ± 0.45 mg rutin/g	NR	[8]
SACE	Acetone	325 ± 2.3 mg rutin/g	8.66 ± 1.9 mg rutin/g	NR	
SAEE	Ethanol	192 ± 3.0 mg rutin/g	7.57 ± 0.09 mg rutin/g	NR	
SAHE	n-hexane	325 ± 2.3 mg rutin/g	5.16 ± 0.9 mg rutin/g	NR	
Leaf extract	Acetone	10.14 ± 0.44 mg tannic acid/g	1.04 ± 0.05 mg QC/g	NR	
Leaf extract	Methanol	10.53 ± 1.29 mg tannic acid/g	0.98 ± 0.10 mg QC/g	NR	
Leaf extract	Water	5.00 ± 0.24 mg tannic acid/g	0.63 ± 0.12 mg QC/g	NR	

CA—catechin; GAE—gallic acid equivalent; MW—microwaves; QC—quercetin; NR—not reported; SAD—non-edible external hard leaves; eu—eudragit; SAME—methanol fraction; SACE—acetone fraction; SAEE—ethanol fraction; SAHE—n-hexane fraction; TAC—total antioxidant capacity; TE—trolox; TFC—total flavonoid content; TPC—total phenol content; U—ultrasound. Values are expressed according to the methodology used in individual studies.

**Table 4:** Antimicrobial spectrum of *Sonchus asper* extracts against skin-relevant pathogens.

	Part of the Plant	Extract	Bacteria	Minimum Inhibitory Concentration (mg/mL)	References
1.	Leaves	Methanol, Ethanol, Water	*S. aureus*, *B. spizizinii*, *E.coli*, *P. aeruginosa*	15	[35]
2.	Whole plant (leaves, stem, flowers, seeds and roots)	Methanolic, n-hexane, ethyl acetate, chloroform, butanolic, water	*S. aureus*, *E.coli*, *K. pneumoniae*, *M. luteus*, *B. subtilis*, *P. aeruginosa*	1	[42]
3.	Leaves	Methanol, Water	*S. aureus*, *B. cerus*, *K. pneumoniae*, *E. coli*	10	[47]
4.	The aerial parts	Methanol	*E. coli* *S. enterica* *S. aureus* *V. parahaemolyticus*	0.04	[36]
5	Leaves	Acetone, methanol, water	*B. cereus*, *S. epidermidis*, *S. aureus*, *M. kristinae*, *S. pyogenes*, *E. coli*, *S. pooni*, *S. marcescens*, *P. aeruginosa*, *K. pneumonae*	2	[38]
6	no data	n-Hexane, Chloroform, Methanol	*S. flexneri*, *Micrococcus* sp., *E. coli*, and *S. aureus*	0.75	[37]

## Data Availability

No new data were created or analyzed in this study. Data sharing is not applicable to this article.

## Supplementary Materials

The following supporting information can be downloaded at: https://www.mdpi.com/article/10.3390/ph19060890/s1. Table S1. Selected marker compounds proposed for standardization of *Sonchus asper* cosmetic raw materials. Table S2. Overview of biological activities of *Sonchus asper* and experimental models used.
