# Supplementary material for: Sonchus asper and Its Potential in Cosmetics—A Review
Source: Pharmaceuticals (Basel). 2026 Jun 4;19(6):890. doi: 10.3390/ph19060890 (PMC13305031; doi:10.3390/ph19060890)
Supplement: Supplementary file 1 [file pharmaceuticals-19-00890-s001.zip › pharmaceuticals-4326241-supplementary.pdf]

Review

Sonchus asper and its potential in cosmetics – a review

Dorota Kasprzak[1], Natalia Dycha[2,3], Magdalena Michalak-Tomczyk[4], Anna Wawruszak[5], Magdalena Zdziebło[6], Wirginia Kukula-Koch[2]*, Grażyna Ginalska[1]

Table S1. Selected marker compounds proposed for standardization of *Sonchus asper* cosmetic raw materials.

Marker compound	Chemical class	Biological relevance	Role in standardization	Analytical method
Chlorogenic acid	Phenolic acid	Strong antioxidant, ROS scavenging	Primary phenolic marker for extract consistency	LC-UV/LC-MS
Caffeic acid	Phenolic acid	Anti-inflammatory, antioxidant activity	Phenolic QC marker	LC-UV
Luteolin-7-glucoside	Flavonoid	Anti-inflammatory, antioxidant	Bioactivity-related marker compound	LC-UV/LC-MS
Apigenin-7-glucoside	Flavonoid	Anti-inflammatory, enzyme modulation	Functional flavonoid marker	LC-UV
Quercetin derivatives	Flavonoid	Broad antioxidant and cytoprotective effects	Flavonoid profile marker	LC-UV / LC–MS
Urospermal A-type derivatives	Sesquiterpene lactones	Anti-inflammatory activity	Secondary phytochemical marker for profiling purposes	LC–MS/MS
Linoleic acid (C18:2), α-linolenic acid (C18:3)	Fatty acids	Skin barrier support, anti-inflammatory potential	Lipid quality marker for cosmetic functionality	GC–MS / LC–MS

Table S2. Overview of biological activities of *Sonchus asper* and experimental models used

Biological property	Experimental model	Assay type	Main outcome	Ref.
Antioxidant activity	DPPH, ABTS, FRAP	*In chemico*	High radical scavenging activity	[6,8,9]
Antioxidant activity	OxHLIA, TBARS	*Ex vivo*	Protection against oxidative hemolysis and lipid oxidation	[6]
Anti-inflammatory activity	RAW264.7 macrophages	*In vitro* (cell-based)	↓ NO, IL-6, TNF-α	[30]
Anti-inflammatory activity	Mouse paw oedema	*In vivo*	Reduced oenema	[27]
Antimicrobial activity	MIC assays	*In vitro*	Acitivity mainly against Gram-positive bacteria	[34-42]
Antifungal activity	Growth inhibition assay	*In vitro*	Inhibition of A. niger and R. solani	[42, 48]
Antidiabetic acitivity	SGLP-1 secretion assay	*In vitro* (cell-based)	↑ GLP-1 secretion	[54-56]
Antidiabetic activity	STZ-induced diabetic rats	*In vivo*	↓ blood glucose and oxidative stress	[57]
Cytotoxic/anticancer activity	Melanoma cel assay	*In vitro (*cell-based*)*	Induced differentiation and growth inhibition	[75]
Hepatoprotective activity	CCl_4 rat model	*In vivo*	↓ oxidative damage of the liver	[77]
